# Noblesse Oblige? Social Status and Economic Inequality Maintenance among Politicians

**DOI:** 10.1371/journal.pone.0085293

**Published:** 2014-01-21

**Authors:** Michael W. Kraus, Bennett Callaghan

**Affiliations:** Department of Psychology, University of Illinois, Urbana-Champaign, Champaign, Illinois, United States of America; University of Minnesota, United States of America

## Abstract

Economic inequality is at historically high levels in the United States and is among the most pressing issues facing society. And yet, predicting the behavior of politicians with respect to their support of economic inequality remains a significant challenge. Given that high status individuals tend to conceive of the current structure of society as fair and just, we expected that high status members of the U.S. House of Representatives would be more likely to support economic inequality in their legislative behavior than would their low status counterparts. Results supported this prediction particularly among Democratic members of Congress: Whereas Republicans tended to support legislation increasing economic inequality regardless of their social status, the social status of Democrats – measured in terms of average wealth, race, or gender – was a significant predictor of support for economic inequality. Policy implications of the observed relationship between social status and support for economic inequality are considered.

## Introduction

“the duty of the Man of Wealth… is to consider all surplus revenues which come to him simply as trust funds, which he is called upon to administer, and strictly bound as a matter of duty to administer in the manner which, in his judgment, is best calculated to produce the most beneficial results for the community…” –Andrew Carnegie [1].

The United States is in the midst of unprecedented levels of economic inequality [2]–[4]. These large scale economic disparities place the most strain on those at the bottom of the social hierarchy – poor and working class families [5] – who must contend with increased poverty, unemployment, problems with health and social support, and homelessness [6]. Americans have few options to combat economic inequality, but they can turn to the democratic system to enact social and fiscal policies that protect individuals from growing wealth disparities. Given that political participation is one of the only avenues available for individuals to combat this economic trend, investigations into the factors that predict whether politicians will support the reduction or increase of economic inequality remain an important area of research.

In the present research, we examined the legislative behavior of members of the US House of Representatives. Drawing on recent psychological research suggesting that individuals with high social status are more likely to view the current structure of society as fair and just (e.g., [7]–[9]), we expected that high status politicians would be more likely to support economic inequality in society in their legislative behavior relative to their low status counterparts.

### Social Status and Meritocratic Beliefs

Social status is broadly defined as the rank-based value of individuals, and can be measured by one's leadership role in organizations, by assessing levels of socioeconomic status (SES; e.g., occupation prestige, annual income), or by one's membership in one or more social categories – such as one's race or gender (e.g., [10]–[12]). However social status is measured, most research finds that higher status confers greater benefits than lower status. For example, when compared to high SES individuals, men, and European Americans, lower status individuals (i.e., low SES individuals, women, and African Americans) experience stereotype threat – anxiety about confirming negative stereotypes about their low status group – that impedes their academic performance (e.g., [13]–[15]). In general, individuals belonging to higher status positions in society benefit from greater access to material and social resources, increased workplace opportunities, and reduced discrimination based on their social status [10], [16]. High status individuals also tend to hold public office more than their low status counterparts, and as a result, have unique access to decision-making power on matters related to economic policy and wealth distribution [5].

Status disparities force high status individuals to explain why they hold a potentially unfair advantage in society relative to their low status counterparts. Recent research indicates that when faced with explaining their elevated social positions, high status individuals endorse meritocratic beliefs (e.g., [7], [9], [17]). Specifically, high status individuals, motivated to maintain their elevated social positions and the benefits they bestow, are particularly likely to explain their many social advantages in terms of a fair application of effort, talent, and skill.

Several lines of empirical evidence suggest that high status individuals endorse meritocratic beliefs more than their low status counterparts. For instance, people with higher status are happier when they believe that positive outcomes in society are based on merit [18]–[19] and high-performing members of a group are more likely to advocate dividing resources based solely on merit [20]. In a recent online survey, individuals with higher income and who subjectively ranked themselves higher in the social class hierarchy in society – using rungs of a ladder based on ascending levels of education, income, and occupation status – reported a greater belief that the world is fair and that society's structure is based on merit than did their lower status counterparts [8]. As a final example, Brandt examined the tendency for individuals to legitimize the social system (e.g., trust the government, express confidence in social institutions) in several representative surveys in the US and abroad. The surveys revealed that high income, white, and male participants were more likely to legitimize the social system than were their low income, non-white, and female counterparts [7].

### Social Status and Economic Inequality

High status individuals' tendency to endorse meritocratic beliefs is indicative of the general preference of these individuals to maintain society in its current structure. Andrew Carnegie's [1] assertion that wealthy individuals should use their influence to help the broader community notwithstanding, there is a long history of elites in society engaging in behavior to maintain the status quo: For centuries, high status individuals have used Divine Right to explain their elevated rank or the seizing of others' resources [21]. As well, elite scientists in the 19^th^ century were famous for espousing Social Darwinism – the thesis that some social groups are inherently superior to others – as a way to justify the valuing of certain individuals over others [22]–[23].

Several theories converge on the prediction that members of high status groups will seek to preserve the economic status quo (e.g., [24]): Realistic group conflict theory suggests that members of high status groups in society, who perceive competition over a scarce resource, will tend to aggress and discriminate against low status groups [25]. As well, group position theory suggests that high status individuals naturally see their own social group as more deserving of elevated social positions and actively work to maintain those positions [26]–[27]. Together, this theoretical work lays the foundation for our central prediction: High status individuals will be more likely to endorse legislation that supports economic inequality relative to their lower status counterparts. We have chosen to study legislation related to economic inequality in society because supporting such legislation is a particularly effective means by which high status individuals can maintain the status quo.

Though no study, to our knowledge, has directly tested the link between social status and support for economic inequality in society, several studies are suggestive of this association: In one illustrative study, university students were asked to examine a chart showing historical increases in economic inequality in society and to explain its causes. Students who rated themselves as higher in social class rank on a ladder representing hierarchy at the university tended to endorse less contextual explanations (e.g., discrimination, educational opportunity) for the economic inequality and more explanations focused on individual merits (e.g., hard work, talent) relative to their lower ranking counterparts [2]. That high status individuals believe economic inequality is caused by individual merit, rather than societal dysfunctions, suggests that these individuals would be less likely to support contextual policy interventions aimed at reducing economic inequality. As a second example, male, relative to female, respondents to the General Social Survey were more likely to explain the gender wage gap by agreeing with the statement that “men work harder” [28]. In terms of race, non-white participants tend to judge policies that attempt to increase representation of ethnic minority groups in organizations (e.g., Affirmative Action) as more fair, necessary, and personally beneficial than do white participants [29]–[31]. Overall, these studies suggest that individuals of high status tend to support economic inequality at work and in society as a whole more than their lower status counterparts.

### The Present Research

Based on the above conceptual analysis, we examined the legislative behavior of members of the US House of Representatives. We expected that members of Congress who belong to relatively high status groups in society would support economic inequality in their legislation more than would their low status counterparts. We chose to study the US House of Representatives because members of Congress, unlike the general public, can use legislation to directly influence economic inequality in the US. As well, though all members of Congress are likely to be high status members of American society, recent research indicates that local rank comparisons are important in revealing the influence of social status on individual psychological processes (e.g., [10]). For instance, having lower status in one's local community predicts lower levels of life-satisfaction better than national income levels [32]–[33]. Thus, we tested the prediction that relative status differences, even among elite members of society, would predict support for economic inequality. Finally, because members of Congress represent a sample of individuals who are much higher in social status and considerably more wealthy than typical university and national samples (e.g., [34]), the present study provides a unique opportunity to generalize the effects of social status to a group of individuals at the top of society's hierarchy.

By far, the most influential predictor of voting behavior among politicians is party identification (e.g., [35]). Thus, in all of our analyses, we account for political party identification and examine its interaction with social status in shaping patterns of support for economic inequality.

## Method

### Participants

This research involved the use of publically available data for 430 members of the US House of Representatives. The data include 190 Democrats and 240 Republicans. The majority of the sample was male (*n* = 357) and white (*n* = 359). Members of Congress had served an average of 11.85 years in office (*SD* = 9.60). This research was approved by the Institutional Review Board of the University of Illinois, Urbana-Champaign.

### Social Status

The social status of members of the House of Representatives was assessed using three variables: average wealth, race, and gender. For average wealth, estimated average wealth of 423 members of the House of Representatives was collected from the Center for Responsive Politics (CRP; www.opensecrets.org). The CRP estimates average wealth based on the broad ranges of wealth and liabilities that members of Congress are required to report to the US government. Average wealth data was collected from 2009 through 2011 and these wealth estimates from each year were averaged to create a measure of overall wealth (*M* = $5.65 million, *SD* = $28.69 million; *α = *.95). As these means clearly suggest, members of Congress were much wealthier than the average American in 2011 (*Mdn*  = $51,100; www.census.gov).

Status based on race and gender was based on self-reports from the websites of each member of Congress (www.house.gov). For analytic purposes, male and white participants were coded as “1” to indicate membership in high status social categories whereas female and non-white participants were coded as “−1” to indicate membership in relatively low status categories. Interestingly, though gender was not related to wealth, *t*(421)  = 0.10, *ns*, non-white (*M* = $0.48 million) members of Congress were significantly less wealthy than their white (*M* = $6.66 million) counterparts, *t*(421)  = 3.71, *p*<.05.

### Economic Legislative Behavior

We analyzed 13 pieces of legislation, chosen by the Institute of Policy Studies (IPS; www.ips-dc.org), that were sponsored by members of Congress between 2010 and 2012. The IPS is a non-profit organization that studies social and economic issues in the US and globally. Legislation (summarized in Table 1) was chosen by the IPS to appear in the 2012 Inequality Report Card [36] if the main policy changes in each bill would directly increase or decrease economic inequality in society. Legislation that increases economic inequality includes bills that reduce regulation of large businesses or that reduce taxes on wealthy Americans. Legislation that decreases economic inequality includes bills that provide forgiveness for student loans and that increase the minimum wage.

**Table 1:** Summary of legislative bills where sponsorship of the bill indicates either support for, or reduction of, economic inequality in the US.

Bill Name and Summary	Inequality	Sponsors
**The Paying a Fair Share Act:** Also known as the “Buffet Rule,” a minimum tax rate of 30% on all those who earn in excess of $1 million dollars annually	Reduce	72
**Freedom to Invest Act:** Provide businesses who earn profits overseas with a one-year fee-free ”tax holiday”	Support	109
**Death Tax Repeal Permanency Act of 2011:** Eliminate the federal estate tax and provide a $5 million gift tax exemption	Support	217
**Humphrey-Hawkins 21st Century Full Employment and Training Act of 2012:** Provides opportunity grants for low-income workforce investment	Reduce	59
**Fair Minimum Wage Act:** Raise the minimum wage to $9.80 within two years, to be increased afterwards according to inflation	Reduce	112
**American Jobs Act:** Create a $50 billion investment in public work and infrastructure projects	Reduce	100
**Keep American Jobs from Going Down the Drain Act:** Requires federally-funded water and sewage projects to use American-made materials	Reduce	39
Paycheck Fairness Act: Allows employees to share salary information with each other and prevent employer retaliation	Reduce	187
**Call Center Worker and Consumer Protection Act:** Notify government of overseas call center, ineligible for tax breaks	Reduce	138
**Permanently Protecting Tenants at Foreclosure Act:** Makes permanent protection of the rights of tenants facing foreclosure	Reduce	24
**Half in Ten Act:** Creates a working group on poverty reduction which would be tasked with developing a 10-year plan to reduce poverty	Reduce	66
**Stephanie Tubbs Jones Assets for Independence Reauthorization Act:** Revises a law providing funding for low-income communities	Reduce	38
**United States National Health Insurance Act:** Provides free health insurance for medically necessary procedures paid for by tax hikes on wealthy	Reduce	71

We examined legislation that has been sponsored by members of Congress, but not yet put to a vote. When members of Congress sponsor legislation, their name is tied to the bill when it is voted on or reported to the public, and thus, sponsoring is particularly indicative of each politician's support for a specific policy. Moreover, whereas decisions about whether to vote on legislation are influenced by a number of factors, including which party has majority control over the House of Representatives, sponsoring legislation is relatively free of these influences. Each bill had, on average, about 95 members of Congress serving as a sponsor or co-sponsor (*M* = 94.77, *SD* = 55.73; see Table 1).

For the sponsored legislation, if a member of Congress sponsored or co-sponsored the legislation that proposes to increase economic inequality, the bill was coded as a “1” whereas sponsoring legislation that proposes to decrease economic inequality was coded as “−1.” When a member of Congress did not sponsor the legislation it was coded as “0.” The sponsored legislation was summed to create an index of support for economic inequality, with higher numbers indicating greater support for economic inequality (*M* = −1.35, *SD* = 3.43; *α* = .90). In the supplementary materials, we also examined 11 pieces of legislation put to a vote by members of Congress (see Analyses S1 in File S1 for additional analyses; see Table S1 in File S1 for a list of legislation).

## Results

We predicted that high status individuals would be more likely to support economic inequality in their legislative behavior relative to their low status counterparts. To test this hypothesis, we used the status of members of Congress, measured in terms of average wealth, race, and gender, to predict sponsoring legislation that supports or reduces economic inequality in society. As expected, political party affiliation had a large effect on sponsoring behavior *t*(428)  = −33.20, *p*<.05 (*M_Dem_*  = −4.62; *M_Rep_*  = 1.25), with Democrats (coded as “−1”) supporting reduction of economic inequality significantly more than Republicans (coded as “1”). All subsequent analyses assess social status predictors of legislative behavior while accounting for party affiliation.

### Average wealth

In the analysis examining average wealth, we conducted a linear regression with standardized average wealth, party affiliation, and their interaction entered as predictors and our index of support for economic inequality entered as the outcome variable. The results aligned with our central prediction: Average wealth emerged as a significant predictor of sponsoring behavior, *β* = .09, *t*(419)  = 2.24, *p*<.05, with wealthier politicians more likely to sponsor legislation that supports economic inequality relative to their poorer counterparts. In this analysis, Republicans were also significantly more likely to sponsor legislation supporting economic inequality than were Democrats, *β* = −.84, *t*(419)  = −32.82, *p*<.05.

**Figure 1:**
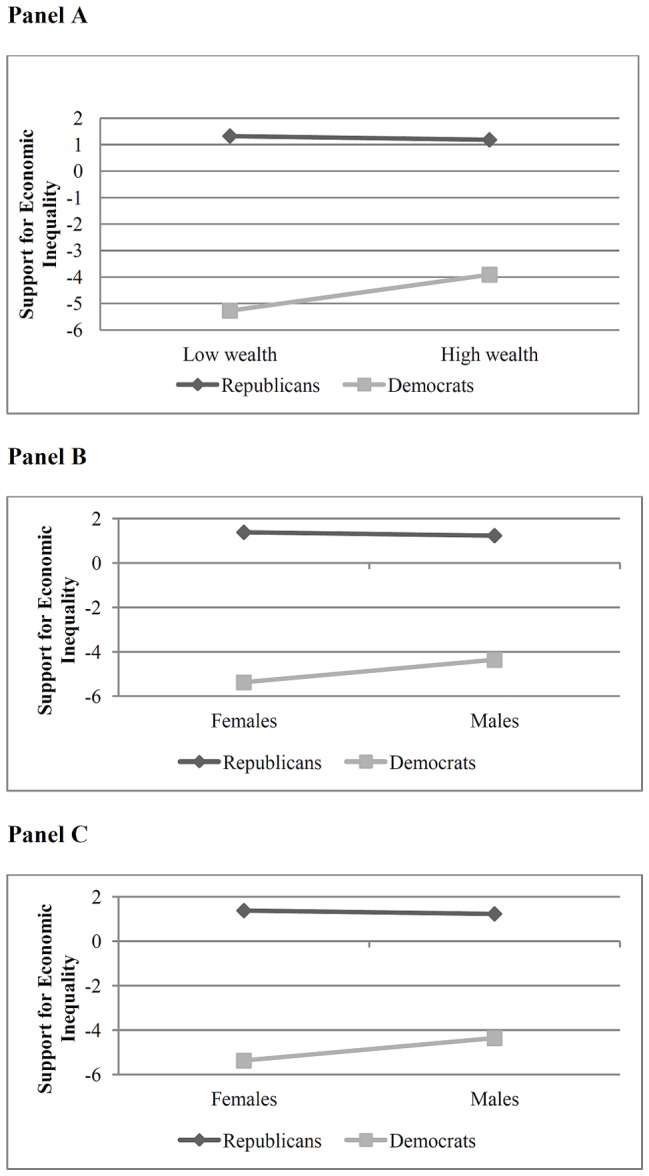
Relationships between social status and the tendency to sponsor legislation supporting economic inequality. Social status is measured in terms of average wealth (Panel A), race (Panel B), and gender (Panel C).

A significant interaction also emerged between party affiliation and average wealth, *β* = .11, *t*(419)  = 2.77, *p*<.05. As shown in Figure 1 (Panel A), whereas Republicans tended to sponsor legislation that supports economic inequality regardless of their wealth, *t*(235)  = −0.45, *ns*, wealth predicted sponsoring behavior for Democrats, *t*(184)  = 4.06, *p*<.05. Specifically, high wealth Democrats tended to sponsor fewer pieces of legislation that reduce economic inequality than did their lower wealth counterparts.

We also conducted a parallel analysis while controlling for the race and gender of members of Congress, to determine if wealth influenced support for economic inequality independent of these other indices of social status. In this analysis, the interaction between wealth and party affiliation was again significant, *β* = .09, *t*(417)  = 2.31, *p*<.05.

### Race

In the analysis examining race, a 2 (race) X 2 (party affiliation) Analysis of Variance (ANOVA) was conducted examining our index of support for economic inequality. Consistent with our central hypothesis, the analysis yielded a main effect for race such that white members of Congress (*M* = −1.34) were more likely to sponsor legislation that supports economic inequality than were their non-white counterparts (*M* = −2.42), *F*(1,426)  = 13.68, *p*<.05. As in the average wealth analysis, a significant main effect of party affiliation also emerged, *F*(1,426)  = 461.65, *p*<.05, as well as a significant interaction, *F*(1,426)  = 14.52, *p*<.05, η_p_^2^ = .03.

Examination of the means revealed a pattern aligning with the average wealth analysis (see Figure 1, Panel B): For Republicans, both white (*M* = 1.24; *CI 95%* [1.02 to 1.46]) and non-white (*M* = 1.27; *CI 95%* [0.27 to 2.27]) members of Congress were equally likely to sponsor legislation supporting economic inequality. For Democrats, white members of Congress (*M* = −3.92; *CI 95%* [−4.21 to −3.63]) were less likely to sponsor legislation reducing economic inequality than were their non-white counterparts (*M* = −6.12; *CI 95%* [−6.55 to −5.69]).

We also examined a parallel 2 (race) X 2 (party affiliation) Analysis of Covariance (ANCOVA) with gender and wealth entered as covariates to determine the unique influence of race on support for economic inequality. The analysis revealed the same significant interaction between race and party affiliation, *F*(1,424)  = 12.32, *p*<.05, η_p_^2^ = .03.

### Gender

In the analysis examining gender, a 2 (gender) X 2 (party affiliation) ANOVA was conducted examining our index of support for economic inequality. The analysis yielded a non-significant effect for gender, *F*(1,426)  = 3.14, *p* = .08, and a significant effect for party affiliation, *F*(1,426)  = 637.54, *p*<.05. These effects were all qualified by a significant interaction, *F*(1,426)  = 5.66, *p*<.05, η_p_^2^ = .01. Examination of the means revealed a pattern aligning with the prior analyses (see Figure 1, Panel C): For Republicans, both male (*M* = 1.23; *CI 95%* [0.99 to 1.47]) and female (*M* = 1.38; *CI 95%* [0.65 to 2.10]) members of Congress were equally likely to sponsor legislation supporting economic inequality. For Democrats, male members of Congress (*M* = −4.36; *CI 95%* [−4.65 to −4.06]) were less likely to sponsor legislation reducing economic inequality than were their female counterparts (*M* = −5.37; *CI 95%* [−5.87 to −4.86]).

We also examined a parallel 2 (gender) X 2 (party affiliation) ANCOVA with race and average wealth entered as covariates to determine the unique influence of gender on support for economic inequality. The analysis revealed the same significant interaction between gender and party affiliation, *F*(1,424)  = 3.73, *p* = .05, η_p_^2^ = .01.

Overall, the above analyses provide support for our central prediction: Individuals with higher social status, measured in terms of average wealth, race, and gender, tend to sponsor legislation that supports economic inequality more than do their lower status counterparts. Importantly, this effect emerged consistently among Democratic members of Congress: Whereas Republicans were uniformly more likely to sponsor legislation that supports economic inequality, regardless of their social status, higher status Democrats were less likely to support legislation reducing economic inequality – such as by sponsoring legislation that forgives student loans or increases the minimum wage – than their low status counterparts.

## Discussion

Belonging to groups of elevated social status has many direct benefits that include access to material and social resources, avoidance of social threats, increased exposure to social and economic opportunities, and influence over economic policy and wealth distribution. We theorized that explaining such widespread benefits forces high status individuals to engage in justifying behavior in favor of the status quo. Thus, we predicted that, relative to lower status individuals, high status individuals would favor legislative behaviors that reinforce their elite status in society – including sponsoring legislation that supports economic inequality. Data on the legislative behavior of members of the US House of Representatives supports this prediction, particularly among Democratic members of Congress. Specifically, high status Democrats tended to exhibit less support for legislation that reduces economic inequality than did their lower status counterparts. In contrast, status did not influence the tendency for Republicans to support economic inequality in their legislative behavior.

It is interesting to speculate about the reasons why status did not influence support for economic inequality among Republicans. One perspective suggests that people who identify as liberal and conservative tend to operate using distinct moral foundations [37]. For instance, people who self-identify as more politically conservative tend to value loyalty and in-group cooperation more than their more liberal counterparts [37]. It is perhaps because of this loyalty that low status members of the Republican Party tended to support economic inequality as much as their high status counterparts. It might also be the case that the Republican Party lacks diversity in Congress: Though the two political parties did not differ in terms of average wealth, *t*(421)  = −1.46, *p* = .14, there were very small numbers of Republicans who identified as non-white (*n* = 11) and female (*n* = 24), suggesting that conclusions about the relations between status and support for economic inequality among these individuals should be viewed as preliminary. Future research would benefit from an examination of individual differences in economic inequality attitudes, particularly among individuals who identify as politically conservative.

It is noteworthy that consistent relationships between support for economic inequality and social status emerged across three distinct measures of social status – average wealth, race, and gender. Importantly, influences of wealth, race, and gender have demonstrated some converging effects in prior research. As we noted above, stereotype threat effects show consistent patterns across SES, race, and gender (e.g., [13]). As a second example, low status in terms of gender and race promotes more interdependent norms for relating to others [38]–[39] as it does in individuals of lower SES [16], [34].

These consistent effects do not mean, however, that all status-related constructs have similar effects across psychological domains. For instance, SES, and wealth in particular, is much more malleable than one's race or gender [11]. This malleability brings up some interesting questions related to the influence of average wealth on support for economic inequality. Specifically, would people who gain in wealth over time be more likely to support economic inequality than would those who were consistently high in wealth across the life course? It is possible that increases in wealth over time enhance beliefs that society is fair and meritocratic – given individuals' knowledge of their own personal struggles and successes – and as a result, promote support for economic inequality. In contrast, being consistently wealthy may give individuals the opportunity to realize the contextual influences on their elevated status in society. Future research would do well to test these predictions.

It is also interesting that social status predicted the legislative behavior of members of Congress, particularly because these individuals are elite members of society with actual influence over social and economic policy. The data presented in this study suggest that despite their membership in an influential social group – the US House of Representatives – individuals still experience their lower status *relative* to other members of the social groups they belong to. The observed pattern of results aligns with a recent body of research suggesting that local status is a primary influence on how individuals conceive of their position in society and relate to others (e.g., [32], [40]).

Finally, the present research also aligns with mounting evidence suggesting that an individual's social status is a reliable predictor of support for economic inequality in society [2], [7]. That social status predicts support for economic inequality among members of Congress – individuals with direct access to creating and implementing policies that shape the future of economic inequality in the US – is a potentially important piece of information for US citizens to consider in future elections. As economic inequality continues to rise in the US [4], and maintains its association with many of the health and social problems facing American society [6], individuals who are particularly likely to seek a reduction in such inequality may be among the most important political leaders of the future.

## Supporting Information

File S1 **Analyses S1.** Supporting statistical analyses containing tests of (1) potential non-linear relationships between variables of interest, and (2) relationships between average wealth, race, and gender and economic inequality voting behavior. **Table** **S1.** Summary of legislative bills such that a yes vote on the bill indicates either support for or reduction of economic inequality in the US.
(DOCX)
