# Correction: Noblesse Oblige? Social Status and Economic Inequality Maintenance Among Politicians

**DOI:** 10.1371/journal.pone.0117892

**Published:** 2015-02-03

**Authors:** The PLOS ONE Staff

In Fig. 1, “Relationships between social status and the tendency to sponsor legislation supporting economic inequality”, Panel B is incorrect. Please see the corrected Fig. 1 here.

**Figure 1:**
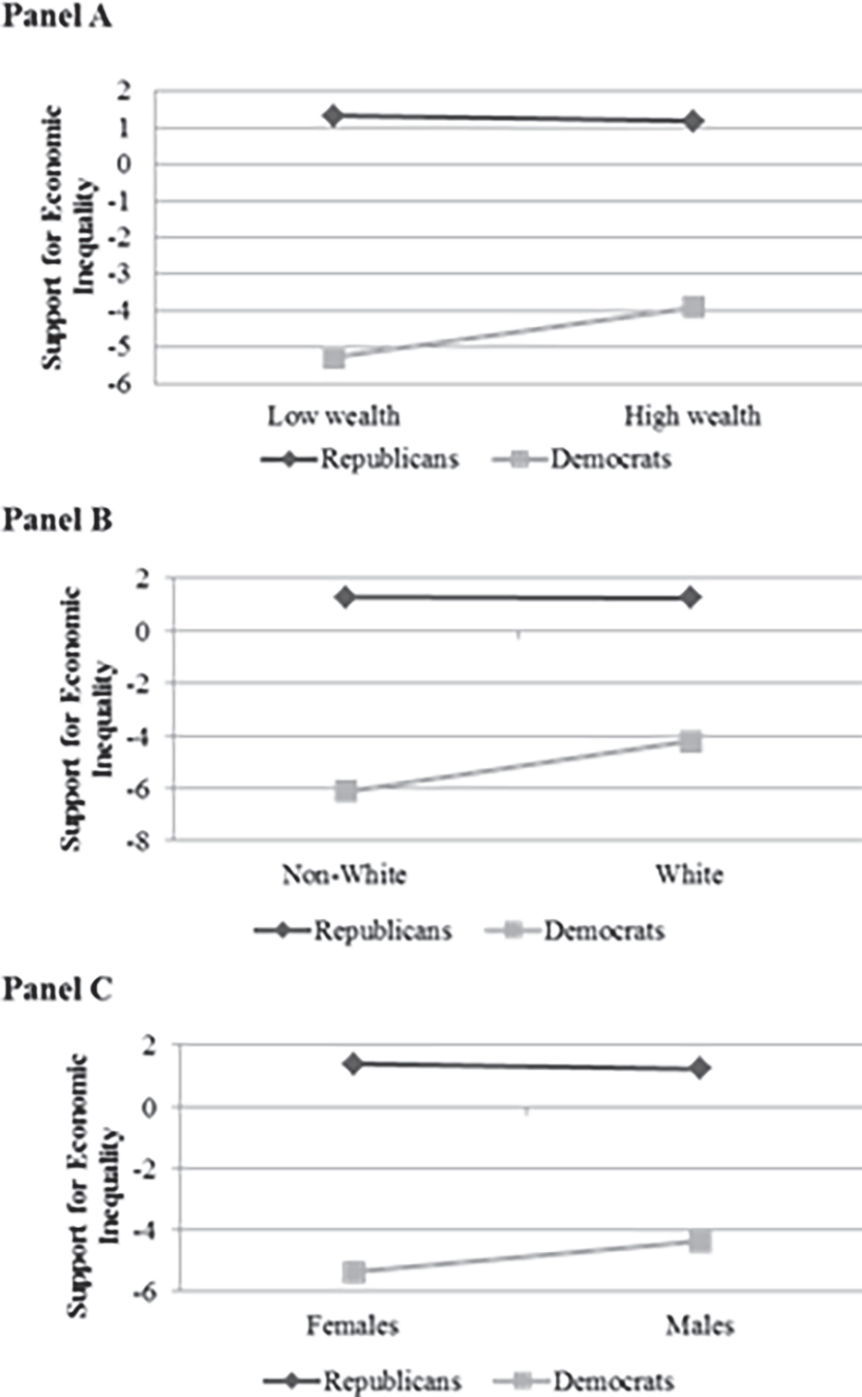
Relationships between social status and the tendency to sponsor legislation supporting economic inequality. Social status is measured in terms of average wealth (Panel A), race (Panel B), and gender (Panel C).
