# Supplementary material for: Noblesse Oblige? Social Status and Economic Inequality Maintenance among Politicians
Source: PLoS One. 2014 Jan 21;9(1):e85293. doi: 10.1371/journal.pone.0085293 (PMC3897426; doi:10.1371/journal.pone.0085293)
Supplement: File S1 — Analyses S1. Supporting statistical analyses containing tests of (1) potential non-linear relationships between variables of interest, and (2) relationships between average wealth, race, and gender and economic inequality voting behavior. Table S1. Summary of legislative bills such that a yes vote on the bill indicates either support for or reduction of economic inequality in the US. (DOCX) [file pone.0085293.s001.docx]

**File S1**

**Noblesse Oblige? Social Status and Economic Inequality Maintenance Among Politicians**

Michael W. Kraus

Department of Psychology

University of Illinois, Urbana-Champaign

Champaign, IL USA

Bennett Callaghan

Department of Psychology

University of Illinois, Urbana-Champaign

Champaign, IL USA

**Analyses S1**

When examining the wealth data, we located two data points with extremely high average wealth (greater than five standard deviations above the mean). When we removed these two outliers, the significant interaction between average wealth and party affiliation on our index of sponsoring behavior in support of economic inequality remained, *β* = .06, *t*(416) = 2.08, *p* < .05. In this analysis, party affiliation was significantly related to sponsoring behavior, *β* = -.84, *t*(416) = -32.53, *p* < .05, whereas average wealth was not, *β* = .03, *t*(416) = 1.25, *p* = .21.

We examined potential non-linear relationships between average wealth and support for economic inequality. In this analysis, we conducted a linear regression predicting sponsoring behavior in support of economic inequality with average wealth, squared wealth, political party, and interactions between all three of these variables. In this analysis, only political party emerged as a significant predictor of sponsoring behavior, *β* = -.83, *t*(416) = -27.99, *p* < .05. Squared wealth was not significantly associated with sponsoring of economic inequality, *β* = -.13, *t*(416) = -0.46, *ns*. This analysis suggests there was no quadratic relationship between wealth and sponsoring of economic inequality legislation.

We also examined voting behavior of members of Congress for legislation related to economic inequality identified by the Institute for Policy Studies. For the vote legislation, we examined 11 pieces of legislation, put to a vote in Congress, that either directly supports or reduces economic inequality (see Table S1, for a summary of the vote legislation). These pieces of vote legislation were coded so that yes or no votes that increase economic inequality were coded as “1” whereas votes that reduce economic inequality were coded as “-1.” When members of Congress did not vote on the legislation, this was coded as “0.” The vote legislation (*M* = 2.40, *SD* = 8.50; *α* = .96) was summed to create an index of support for economic inequality, with higher numbers indicating greater support for economic inequality.

In the analysis examining the voting behavior index and average wealth, only party affiliation emerged as a significant predictor, with Republicans more likely to vote in favor of legislation that supports economic inequality than Democrats, *β* = -.96, *t*(419) = -66.34, *p* < .05. Average wealth and the interaction between wealth and party affiliation were not related to voting behavior in this analysis (*ts* < 1).

For race, significant main effects for race, *F*(1,426) = 10.66, *p* < .05, and party affiliation, *F*(1,426) = 1485.45, *p* < .05, emerged. White (*M* = 1.77) and Republican (*M* = 9.24) members of Congress were more likely to vote for legislation that supports economic inequality than were non-white (*M* = 0.39) and Democratic (*M* = -7.07) members, respectively. The interaction was not significant in this analysis, *F*(1,426) = 1.66, *p* = .20.

For gender, the non-significant main effect for gender, *F*(1,426) < 1, and the significant effect for party affiliation, *F*(1,426) = 2449.60, *p* < .05, were qualified by a significant interaction *F*(1,426) = 5.54, *p* < .05: For Republicans, both male (*M* = 9.57; *CI 95%* [9.24 to 9.90]) and female (*M* = 10.04; *CI 95%* [9.04 to 11.04]) members of Congress were equally likely to vote for legislation supporting economic inequality. For Democrats, male members of Congress (*M* = -6.43; *CI 95%* [-6.84 to -6.01]) were less likely to vote for legislation reducing economic inequality than were their female counterparts (*M* = -7.55; *CI 95%* [-8.25 to -6.85]).

| **Table S1.** | | | |
| --- | --- | --- | --- |
| **Bill Name and Summary** | **Inequality** | **Yes Votes** | **No Votes** |
| **Job Protection and Recession Prevention Act:** Extends the “Bush Tax Cuts” | Support | 256 | 171 |
| **Doggett Amendment to Stop Tax Haven Abuse Act:** Higher penalties for tax haven abuse, and gives federal agents more authority to investigate | Reduce | 192 | 223 |
| **Small Business Tax Cut Act:** Small businesses deduct 20% of active income from taxes | Support | 233 | 172 |
| **Paul Ryan Budget:** Proposal to balance federal budget by cutting spending and entitlements | Support | 232 | 188 |
| **People’s Budget Amendment to Resolution 112:** Proposal to reduce deficit by eliminating tax cuts on wealthy and corporations | Reduce | 78 | 343 |
| **Sequester Replacement Reconciliation Act of 2012:** Replace military spending cuts with cuts to entitlement programs | Support | 216 | 198 |
| **Jumpstart Our Business Startups Act:** Reduces regulation of business by the Securities and Exchange Commission | Support | 377 | 41 |
| **Amendment to Eliminate Prevailing Wage Provisions of Davis-Bacon Act:** Repeals the Davis-Bacon Act of 1931, which dictates that prevailing local wages be paid for public work acts | Support | 192 | 222 |
| **Protecting Jobs from Government Interference Act:** Reduces authority of the National Labor Relations Board (NLRB) to regulate business | Support | 263 | 183 |
| **Interest Rate Reduction Act:** Prevent the federal student loan interest rate from rising, funds would be used for government assistance programs | Support | 213 | 195 |
| **Kline Amendment to Resolution 1:** Prevents educational institutions’ access to federal college loans if their students graduate with high levels of default | Support | 284 | 133 |
